# Supplementary material for: In Vitro and In Vivo Human Metabolism of Ostarine, a Selective Androgen Receptor Modulator and Doping Agent
Source: Int J Mol Sci. 2024 Jul 17;25(14):7807. doi: 10.3390/ijms25147807 (PMC11277069; doi:10.3390/ijms25147807)
Supplement: Supplementary file 1 [file ijms-25-07807-s001.zip › Taoussi-OstaMetID_TableS1-InSil_FINAL.pdf]

Table S1. Ostarine putative metabolites predicted with GLORYx freeware and their prediction score (adjusted score for second-generation metabolites)

ID	Transformation	Elemental composition	Score	Simplified molecular-input line-entry system (SMILES)
pA1	*O*-Glucuronidation	$C_{25}H_{22}F_3N_3O_9$	78%	O=C(Nc1cc(c(cc1)C#N)C(F)(F)F)C(C)(OC1OC(C(O)=O)C(O)C(O)C1O)COc1ccc(C#N)cc1
pA1-1	+ Glutathione Conjugation (nitrile)	$C_{35}H_{39}F_3N_6O_{15}S$	39%	O=C(Nc1ccc(C#N)c(c1)C(F)(F)F)C(C)(OC1OC(C(O)=O)C(O)C(O)C1O)COc1ccc(cc1)C(=N)SCC(NC(=O)CCC(N)C(O)=O)C(=O)NCC(O)=O
pA1-2	+ Glutathione Conjugation (nitrile)	$C_{35}H_{39}F_3N_6O_{15}S$	35%	O=C(Nc1ccc(C(=N)SCC(NC(=O)CCC(N)C(O)=O)C(=O)NCC(O)=O)c(c1)C(F)(F)F)C(C)(OC1OC(C(O)=O)C(O)C(O)C1O)COc1ccc(cc1)C#N
pA2	Hydrolysis (secondary amide)	$C_{11}H_{11}NO_4$	69%	OC(C)(COc1ccc(C#N)cc1)C(O)=O
pA2-1	+ *O*-Glucuronidation (aliphatic hydroxyl)	$C_{17}H_{19}NO_{10}$	62%	OC(=O)C(C)(COc1ccc(C#N)cc1)OC1OC(C(O)=O)C(O)C(O)C1O
pA2-2	+ *O*-Glucuronidation (aliphatic carboxyl)	$C_{17}H_{19}NO_{10}$	37%	OC(C)(COc1ccc(C#N)cc1)C(=O)OC1OC(C(O)=O)C(O)C(O)C1O
pA2-3	+ Glutathione Conjugation (nitrile)	$C_{21}H_{28}N_4O_{10}S$	33%	OC(C)(COc1ccc(cc1)C(=N)SCC(NC(=O)CCC(N)C(O)=O)C(=O)NCC(O)=O)C(O)=O
pA2-4	+ Decarboxylation	$C_{10}H_{11}NO_2$	31%	OC(C)COc1ccc(C#N)cc1
pA3	Hydrolysis (secondary amide)	$C_8H_5F_3N_2$	69%	FC(F)(F)c1cc(N)ccc1C#N
pA3-1	+ *N*-acetylation (aniline)	$C_{10}H_7F_3N_2O$	66%	O=C(C)Nc1cc(c(C#N)cc1)C(F)(F)F
pA3-2	+ Sulfation (aniline)	$C_8H_5F_3N_2O_3S$	65%	FC(F)(F)c1cc(ccc1C#N)NS(O)(=O)=O
pA3-3	+ *N*-Glucuronidation (aniline)	$C_{14}H_{13}F_3N_2O_6$	65%	OC(=O)C1OC(Nc2cc(c(C#N)cc2)C(F)(F)F)C(O)C(O)C1O
pA3-4	+ *N*-Oxidation (aniline)	$C_8H_5F_3N_2O$	31%	FC(F)(F)c1cc(ccc1C#N)NO
pA3-5	+ Aromatic Hydroxylation (ortho to 2 substituents)	$C_8H_5F_3N_2O$	31%	FC(F)(F)c1c(O)c(N)ccc1C#N
pA3-6	+ Glutathione Conjugation (nitrile)	$C_{18}H_{22}F_3N_5O_6S$	29%	OC(=O)C(N)CCC(=O)NC(CSC(=N)c1ccc(N)cc1C(F)(F)F)C(=O)NCC(O)=O
pA4	Amine Hydroxylation	$C_{19}H_{14}F_3N_3O_4$	69%	ON(C(=O)C(O)(C)COc1ccc(C#N)cc1)c1cc(c(cc1)C#N)C(F)(F)F
pA4-1	+ *N*-Oxidation	$C_{19}H_{14}F_3N_3O_5$	36%	[O-][N+](O)(C(=O)C(O)(C)COc1ccc(C#N)cc1)c1cc(c(cc1)C#N)C(F)(F)F
pA4-2	+ Hydrolysis (heteroatom bonded amide)	$C_{11}H_{11}NO_4$	36%	OC(C)(COc1ccc(C#N)cc1)C(O)=O
pA4-3	+ Hydrolysis (heteroatom bonded amide)	$C_8H_5F_3N_2O$	36%	FC(F)(F)c1cc(ccc1C#N)NO
pA4-4	+ Glutathione Conjugation (nitrile)	$C_{29}H_{31}F_3N_6O_{10}S$	35%	ON(C(=O)C(O)(C)COc1ccc(cc1)C(=N)SCC(NC(=O)CCC(N)C(O)=O)C(=O)NCC(O)=O)c1ccc(C#N)c(c1)C(F)(F)F
pA4-5	+ Glutathione Conjugation (nitrile)	$C_{29}H_{31}F_3N_6O_{10}S$	31%	ON(C(=O)C(O)(C)COc1ccc(cc1)C#N)c1ccc(C(=N)SCC(NC(=O)CCC(N)C(O)=O)C(=O)NCC(O)=O)c(c1)C(F)(F)F
pA5	Glutathione Conjugation (nitrile)	$C_{29}H_{31}F_3N_6O_9S$	51%	OC(C)(COc1ccc(cc1)C(=N)SCC(NC(=O)CCC(N)C(O)=O)C(=O)NCC(O)=O)C(=O)Nc1ccc(C#N)c(c1)C(F)(F)F
pA5-1	+ *O*-Glucuronidation (aliphatic carboxyl)	$C_{35}H_{39}F_3N_6O_{15}S$	46%	OC(C)(COc1ccc(cc1)C(=N)SCC(NC(=O)CCC(N)C(=O)OC1OC(C(O)=O)C(O)C(O)C1O)C(=O)NCC(O)=O)C(=O)Nc1ccc(C#N)c(c1)C(F)(F)F
pA5-2	+ *N*-Acetylation (aliphatic NH2)	$C_{31}H_{33}F_3N_6O_{10}S$	45%	OC(C)(COc1ccc(cc1)C(=N)SCC(NC(=O)CCC(NC(=O)C)C(O)=O)C(=O)NCC(O)=O)C(=O)Nc1ccc(C#N)c(c1)C(F)(F)F
pA5-3	+ *O*-Glucuronidation (aliphatic carboxyl)	$C_{35}H_{39}F_3N_6O_{15}S$	40%	OC(C)(COc1ccc(cc1)C(=N)SCC(NC(=O)CCC(N)C(O)=O)C(=O)NCC(=O)OC1OC(C(O)=O)C(O)C(O)C1O)C(=O)Nc1ccc(C#N)c(c1)C(F)(F)F
pA5-4	+ *O*-Glucuronidation (aliphatic hydroxyl)	$C_{35}H_{39}F_3N_6O_{15}S$	30%	O=C(Nc1ccc(C#N)c(c1)C(F)(F)F)C(C)(OC1OC(C(O)=O)C(O)C(O)C1O)COc1ccc(cc1)C(=N)SCC(NC(=O)CCC(N)C(O)=O)C(=O)NCC(O)=O
pA6	Glutathione Conjugation (nitrile)	$C_{29}H_{31}F_3N_6O_9S$	45%	OC(C)(COc1ccc(cc1)C#N)C(=O)Nc1ccc(C(=N)SCC(NC(=O)CCC(N)C(O)=O)C(=O)NCC(O)=O)c(c1)C(F)(F)F
pA6-1	+ *O*-Glucuronidation (aliphatic carboxyl)	$C_{35}H_{39}F_3N_6O_{15}S$	41%	OC(C)(COc1ccc(cc1)C#N)C(=O)Nc1ccc(C(=N)SCC(NC(=O)CCC(N)C(=O)OC2OC(C(O)=O)C(O)C(O)C2O)C(=O)NCC(O)=O)c(c1)C(F)(F)F
pA6-2	+ *N*-Acetylation (aliphatic NH2)	$C_{35}H_{39}F_3N_6O_{15}S$	40%	OC(C)(COc1ccc(cc1)C#N)C(=O)Nc1ccc(C(=N)SCC(NC(=O)CCC(NC(=O)C)C(O)=O)C(=O)NCC(O)=O)c(c1)C(F)(F)F
pA6-3	+ *O*-Glucuronidation (aliphatic carboxyl)	$C_{35}H_{39}F_3N_6O_{15}S$	36%	OC(C)(COc1ccc(cc1)C#N)C(=O)Nc1ccc(C(=N)SCC(NC(=O)CCC(N)C(O)=O)C(=O)NCC(=O)OC2OC(C(O)=O)C(O)C(O)C2O)c(c1)C(F)(F)F
pA6-4	+ *O*-Glucuronidation (aliphatic hydroxyl)	$C_{35}H_{39}F_3N_6O_{15}S$	27%	O=C(Nc1ccc(C(=N)SCC(NC(=O)CCC(N)C(O)=O)C(=O)NCC(O)=O)c(c1)C(F)(F)F)C(C)(OC1OC(C(O)=O)C(O)C(O)C1O)COc1ccc(cc1)C#N
pA7	*O*-Dealkylation (aromatic)	$C_{12}H_{11}F_3N_2O_3$	35%	OC(C)(CO)C(=O)Nc1cc(c(C#N)cc1)C(F)(F)F
pA7-1	+ *O*-Glucuronidation (aliphatic hydroxyl)	$C_{18}H_{19}F_3N_2O_9$	28%	O=C(Nc1cc(c(C#N)cc1)C(F)(F)F)C(C)(CO)OC1OC(C(O)=O)C(O)C(O)C1O
pA7-2	+ *O*-Glucuronidation (aliphatic hydroxyl)	$C_{18}H_{19}F_3N_2O_9$	25%	OC(C)(COC1OC(C(O)=O)C(O)C(O)C1O)C(=O)Nc1cc(c(C#N)cc1)C(F)(F)F
pA8	Oxidative ether cleavage to one alcohol and one aldehyde/ketone	$C_{12}H_9F_3N_2O_3$	35%	OC(C)(C=O)C(=O)Nc1cc(c(C#N)cc1)C(F)(F)F
pA8-1	+ *O*-Glucuronidation (aliphatic hydroxyl)	$C_{18}H_{17}F_3N_2O_9$	25%	O=C(Nc1cc(c(C#N)cc1)C(F)(F)F)C(C)(C=O)OC1OC(C(O)=O)C(O)C(O)C1O
pA8-2	+ Sulfation (aliphatic hydroxyl)	$C_{12}H_9F_3N_2O_6S$	24%	OS(=O)(=O)OC(C)(C=O)C(=O)Nc1cc(c(C#N)cc1)C(F)(F)F
pA9	Dehydration next to SP2 a	$C_{19}H_{12}F_3N_3O_2$	35%	O=C(Nc1cc(c(cc1)C#N)C(F)(F)F)/C(C)=C/Oc1ccc(C#N)cc1
pA9-1	+ Glutathione Conjugation (alpha,beta-unsaturated carbonyl)	$C_{29}H_{29}F_3N_6O_8S$	22%	OC(=O)C(N)CCC(=O)NC(CSC(Oc1ccc(cc1)C#N)C(C)C(=O)Nc1ccc(C#N)c(c1)C(F)(F)F)C(=O)NCC(O)=O
pA10	*O*-Dealkylation (aromatic)	C_7H_5NO	35%	Oc1ccc(C#N)cc1
pA10-1	+ Sulfation (aromatic hydroxyl)	$C_7H_5NO_4S$	35%	O=S(O)(=O)Oc1ccc(C#N)cc1
pA10-2	+ *O*-Glucuronidation (aromatic hydroxyl)	$C_{13}H_{13}NO_7$	34%	OC(=O)C1OC(Oc2ccc(C#N)cc2)C(O)C(O)C1O
pA10-3	+ Aromatic Hydroxylation (ortho to oxygen)	$C_7H_5NO_2$	26%	Oc1ccc(C#N)cc1O
pA11	Aliphatic Hydroxylation	$C_{19}H_{14}F_3N_3O_4$	35%	OC(C)(C(O)Oc1ccc(C#N)cc1)C(=O)Nc1cc(c(cc1)C#N)C(F)(F)F
pA11-1	+ *O*-Glucuronidation (aliphatic hydroxyl)	$C_{25}H_{22}F_3N_3O_{10}$	26%	OC(Oc1ccc(C#N)cc1)C(C)(OC1OC(C(O)=O)C(O)C(O)C1O)C(=O)Nc1cc(c(cc1)C#N)C(F)(F)F
pA12	Sulfation (aliphatic hydroxyl)	$C_{19}H_{14}F_3N_3O_6S$	29%	OS(=O)(=O)OC(C)(COc1ccc(C#N)cc1)C(=O)Nc1cc(c(cc1)C#N)C(F)(F)F
