# Supplementary material for: In Vitro and In Vivo Human Metabolism of Ostarine, a Selective Androgen Receptor Modulator and Doping Agent
Source: Int J Mol Sci. 2024 Jul 17;25(14):7807. doi: 10.3390/ijms25147807 (PMC11277069; doi:10.3390/ijms25147807)
Supplement: Supplementary file 1 [file ijms-25-07807-s001.zip › Taoussi-OstaMetID_TableS2-InclList_FINAL.pdf]

Table S2. Inclusion list used during liquid chromatography-high-resolution tandem mass spectrometry (LC-HRMS/MS) for ostarine metabolite identification

Transformation	Elemental composition	$[M+H]^+$ *m/z*	$[M-H]^-$ *m/z*
PARENT (Ostarine)	$C_{19}H_{14}F_3N_3O_3$	390.1060	388.0915
+1O	$C_{19}H_{14}F_3N_3O_4$	406.1009	404.0864
-7C-3H-1N	$C_{12}H_{11}F_3N_2O_3$	289.0795	287.0649
-8C-3H-3F-2N+1O	$C_{11}H_{11}NO_4$	222.0761	220.0615
-11C-9H-1N-3O	$C_8H_5F_3N_2$	187.0478	185.0332
+6C+8H+6O	$C_{25}H_{22}F_3N_3O_9$	566.1381	564.1235
+3O+1S	$C_{19}H_{14}F_3N_3O_6S$	470.0628	468.0483
-12C-9H-3F-2N-2O	$C_7H_5N_O$	120.0444	118.0298
+10C+17H+3N+6O+1S	$C_{29}H_{31}F_3N_6O_9S$	697.1898	695.1753
-2H-1O	$C_{19}H_{12}F_3N_3O_2$	372.0954	370.0809
-7C-5H-1N	$C_{12}H_9F_3N_2O_3$	287.0638	285.0493
+6C+8H+7O	$C_{25}H_{22}F_3N_3O_{10}$	582.1330	580.1185
+2O	$C_{19}H_{14}F_3N_3O_5$	422.0958	420.0813
-7C-3H-1N+O	$C_{12}H_{11}F_3N_2O_4$	305.0744	303.0598
-8C-3H-3F-2N+2O	$C_{11}H_{11}NO_5$	238.0710	236.0564
+4O+1S	$C_{19}H_{14}F_3N_3O_7S$	486.0577	484.0432
+10C+17H+3N+7O+1S	$C_{29}H_{31}F_3N_6O_{10}S$	713.1847	711.1702
-11C-9H-1N-2O	$C_8H_5F_3N_2O$	203.0427	201.0281
-2C+5H-3F-2N+7O	$C_{17}H_{19}NO_{10}$	398.1082	396.0936
+2C+14H-3F+1N+7O+1S	$C_{21}H_{28}N_4O_{10}S$	529.1599	527.1453
-9C-3H-3F-2N-1O	$C_{10}H_{11}NO_2$	178.0863	176.0717
-5C-1H-1N+3O	C14H13F3N2O6	363.0798	361.0653
-11C-9H-1N-+S	$C_8H_5F_3N_2O_3S$	267.0046	264.9900
-9C-7H-1N-2O	$C_{10}H_7F_3N_2O$	229.0583	227.0438
-1C+8H+2N+3O+S	$C_{18}H_{22}F_3N_5O_6S$	494.1316	492.1170
-1C+5H-1N+6O	$C_{18}H_{19}F_3N_2O_9$	465.1115	463.0970
-7C-3H-1N+3O+1S	$C_{12}H_{11}F_3N_2O_6S$	369.0363	367.0217
-1C+3H-1N+6O	$C_{18}H_{17}F_3N_2O_9$	463.0959	461.0813
-7C-5H-1N+3O+1S	$C_{12}H_9F_3N_2O_6S$	367.0206	365.0061
-12C-9H-3F-2N-O	$C_7H_5NO_2$	136.0393	134.0248
-6C-1H-3F-2N+4O	$C_{13}H_{13}NO_7$	296.0765	294.0619
-12C-9H-3F-2N+1O+1S	$C_7H_5NO_4S$	200.0012	197.9867
+10C+15H+3N+5O+1S	$C_{29}H_{29}F_3N_6O_8S$	679.1792	677.1647
+5C+10H+2N+3O+1S	$C_{24}H_{24}F_3N_5O_6S$	568.1472	566.1327
+3C+7H+1N+2O+1S	$C_{22}H_{21}F_3N_4O_5S$	511.1258	509.1112
+2H+1S	$C_{19}H_{16}F_3N_3O_3S$	424.0937	422.0792
