# Supplementary material for: In Vitro and In Vivo Human Metabolism of Ostarine, a Selective Androgen Receptor Modulator and Doping Agent
Source: Int J Mol Sci. 2024 Jul 17;25(14):7807. doi: 10.3390/ijms25147807 (PMC11277069; doi:10.3390/ijms25147807)
Supplement: Supplementary file 1 [file ijms-25-07807-s001.zip › Taoussi-OstaMetID_TableS3-CDSett_FINAL.pdf]

**Table S3.** Compound Discoverer processing settings for generating ostarine putative metabolites

| | |
|---|---|
| **Phase I reactions** | Amide hydrolysis (-11C -9H -N -3O → ø)<br>Amide hydrolysis to COOH (-8C -4H -2N -3F → ø)<br>Desaturation (-2H → ø)<br>Dihydrodiol formation (ø → +2H +2O)<br>Ether cleavage 1 (-7C -3H -2N -2O → ø)<br>Ether cleavage 2 (-12C -9H -2N -2O -3F → ø)<br>Ketone formation (O →+2H)<br>Oxidation (ø → +O)<br>Oxidative Deamination to Alcohol (-2H -N → +H +O)<br>Oxidative Deamination to Ketone (-3H -N → +O)<br>Reduction (ø → +2H) |
| **Phase II reactions** | Acetylation (-H → +2C +3H +O)<br>Cysteine conjugation on nitrile (ø → +3C +7H +N +2O +S)<br>Cysteine-Glycine conjugation on nitrile (ø → +5C +10H +2N +3O +S)<br>Glycine conjugation on $NH_2$ (-H → +2C +3H +2O)<br>Glucuronide conjugation (-H → +6C +9H +6O)<br>Glutathione conjugation on nitrile (ø → +10C +17H +3N +6O +S)<br>Methylation (-H → +C +3H)<br>Sulfation (-H → +H +3O +S)<br>Thiol conjugation (-2H -S → ø) |
| **Max number of dealkylations** | 2 |
| **Max number of phase II reactions** | 2 |
| **Max number of all steps** | 5 |
| **Adducts** | $[M+H]^+$<br>$[M-H]^-$ |
